# Multi-Omics Integration Reveals the Impact of Gastrointestinal Microbiota on Feed Efficiency in Tan Sheep

**DOI:** 10.3390/microorganisms13071608

**Published:** 2025-07-08

**Authors:** Guohan Sun, Xiaohong Han, Tonggao Liu, Xinrui Zhang, An Shi, Chong Yang, Jinzhong Tao

**Affiliations:** 1 College of Animal Science and Technology, Ningxia University, Yinchuan 750021, China; sunguohan01@163.com (G.S.); hanxh1254@126.com (X.H.); iszhangxinr@163.com (X.Z.); shian_1988@outlook.com (A.S.); 2 Animal Husbandry Workstation of Ningxia, Yinchuan 750021, China; nxxmztykl@163.com (T.L.); xmjych7203@163.com (C.Y.)

**Keywords:** rumen, cecum, rectum, 16S rRNA, metabolomics

## Abstract

The rumen and intestinal microbiota play a pivotal role in the digestion and absorption processes of ruminants. Elucidating the mechanisms by which gastrointestinal microbiota influence the feed conversion ratio (FCR) in ruminants is significantly important for enhancing feed utilization efficiency in these animals. In this study, RT-qPCR, 16S rRNA sequencing, and metabolomic techniques were systematically employed to compare the microbial community structures in the rumen, cecum, and rectum, as well as the differences in rumen metabolites between high- and low-FCR Tan sheep. The results showed that, compared to the HFCR group of Tan sheep, the LFCR group exhibited a significant reduction in unclassified_f__Selenomonadaceae, Blvii28_wastewater-sludge_group, and Papillibacter in the rumen; a significant increase in Lachnospiraceae_AC2044_group and Sanguibacteroides; a significant reduction in unclassified_f__Peptostreptococcaceae, Clostridium_sensu_stricto_1, and Parasutterella in the cecum; a significant increase in norank_f__Bacteroidales_UCG-001; and a significant reduction in norank_f__Muribaculaceae, Blautia, and Turicibacter in the rectum. There is a significant positive correlation between Parasutterella in the cecum and three microorganisms, including unclassified_f__Selenomonadaceae, in the rumen. Additionally, Blvii28_wastewater-sludge_group was positively correlated with Lactobacillus. Furthermore, unclassified_f__Selenomonadaceae in the rumen was positively correlated with Turicibacter, unclassified_f__Peptostreptococcaceae, and Breznakia in the rectum. Blvii28_wastewater-sludge_group also showed positive correlations with Blautia, norank_f__Muribaculaceae, and Clostridium_sensu_stricto_1, while Papillibacter was positively correlated with Faecalitalea. The metabolomic results indicated that, compared to the HFCR group, 261 differential metabolites, including Phenylacetylglutamine and Populin, in the rumen of Tan sheep in the LFCR group were significantly downregulated, whereas 36 differential metabolites, including Glycyl-L-tyrosine, were significantly upregulated. Furthermore, the rumen microbe unclassified_f__Selenomonadaceae exhibited positive correlations with significantly differential metabolites such as L-tryptophan, Etiocholanolone glucuronide, N-acetyl-O-demethylpuromycin, and 6-deoxyerythronolide B. Blvii28_wastewater-sludge_group and Papillibacter also exhibited positive correlations with Icilin. High and low FCRs in the rumen of Tan sheep were investigated, especially in relation to unclassified_f__Selenomonadaceae, Blvii28_wastewater-sludge_group, and Papillibacter. Correlations can be seen with microorganisms such as Parasutatella and Lactobacillus in the cecum; Turicibacter, norank_f__Bacteroideales_UCG-001, and Blautia in the rectum; and metabolites such as L-tryptophan, Etiocholanolone glucuronide, and N-acetyl-O-demethylpuromycin. This reveals the role of microorganisms in the digestion and absorption of Tan sheep feed, thus providing a preliminary basis for further research on the microbial regulation of ruminant animal feed utilization and a theoretical basis for improving Tan sheep feed utilization efficiency.

## 1. Introduction

The Tan sheep is an essential economic livestock species in the arid regions of northwest China that possesses genetic traits such as tolerance to roughage and strong stress resistance. While Tan sheep can thrive under low-quality forage conditions, significant variations in the FCR can occur during their growth and development [1,2]. The FCR is a critical indicator for assessing the efficiency with which animals convert feed into body weight gain; a lower FCR indicates higher feed utilization efficiency [3,4]. The gastrointestinal microbial community is an ecosystem composed of a diverse array of microorganisms, which significantly impacts the growth and feed utilization efficiency of ruminants [5,6,7]. Recent studies on Hu sheep have shown that an increase in microorganisms such as Prevotella in the rumen can enhance the feed utilization efficiency of the host by synthesizing short-chain fatty acids and essential nutrients [8,9]. Additionally, the cecal and rectal microbiota influence the feed utilization efficiency of sheep through mechanisms such as the secondary degradation of undigested fibers [10,11]. A study by Zhang et al. indicated that the abundance of microbiota, such as Lachnospiraceae, in the cecum is higher in low-FCR ram lambs [12]. In their research on sheep, Kang et al. also demonstrated that those with a high abundance of microbiota, such as Prevotellaceae_UCG-001, in the rectum exhibit higher feed efficiency [13]. Research by Li et al. found that changes in the abundance of microbiota, such as Prevotella in the rumen, mainly affect the feed utilization efficiency of sheep by enriching metabolic pathways, such as the alanine-glutamate pathway [14]. Although current studies have revealed the mechanisms linking the microbial communities in the digestive tract of ruminants and rumen metabolites with the FCR, they mostly focus on single analyses, and there is a lack of systematic research on the impact of gastrointestinal microbiota on feed efficiency in Tan sheep [15,16]. Therefore, this study analyzed the structure of microbial communities in the rumen, cecum, and rectum of high- and low-FCR Tan sheep through 16S rRNA sequencing and conducted correlation analyses between the rumen and the cecum, as well as the rectum. Furthermore, metabolomic technology was employed to explore the differences in rumen metabolites between high- and low-FCR Tan sheep and to analyze the correlations between significantly different metabolites and microorganisms. The aim of this research is to provide a theoretical basis for improving feed utilization efficiency by optimizing the microbial communities in the digestive tract of Tan sheep.

## 2. Materials and Methods

### 2.1. Experimental Animals and Feeding Management

This study was conducted in strict compliance with laws and regulations, and ethical approval was obtained from the Science and Technology Ethics Committee of Ningxia University (Approval No.: NXU-2024-143). The trial site was a Tan sheep farm located in Wuzhong City, Ningxia Hui Autonomous Region (103° E, 35° N), at an altitude of 1600 m. Based on the criteria of sheep having to be a similar age (3 months old) and weight (31.69 ± 3.72 kg), 156 healthy male Tan sheep that met these conditions were selected from this Tan sheep farm. Subsequently, the sheep were randomly divided into four groups and uniformly raised and managed. The sheep were fed twice daily at 07:00 and 18:00 (with feed troughs emptied before feeding), and each feeding session was controlled to last 30 min. All experimental Tan sheep were fed using the neck clamp method. The experimental period was divided into a transition phase of 15 days, a preliminary phase of 10 days, and a formal experimental phase of 50 days. The experimental Tan sheep were weighed every 10 days from the beginning to the end of the formal trial period. Daily feed intake and leftovers were recorded, and daily feed consumption was calculated by subtracting the amount of leftovers from the total feed provided. On the day following the conclusion of the formal trial period, the FCR values of all participating Tan sheep were calculated. The sheep were then ranked based on the average and standard deviation of their FCR values. According to the criteria of FCR > Mean + 0.5 SD and FCR < Mean − 0.5 SD, the participating Tan sheep were divided into a high-FCR group (HFCR group) and a low-FCR group (LFCR group). Five sheep were randomly selected from each group, for a total of 10 Tan sheep. The dietary information for all participating Tan sheep is presented in Table 1.

### 2.2. Experimental Sample Collection

On the morning following the completion of the trial period, rumen, cecum, and rectal contents were collected from the 10 Tan sheep in both the HFCR and LFCR groups. These samples were aliquoted into 5 mL cryotubes and stored at −80 °C for subsequent experimental research.

### 2.3. 16S rRNA Sequence Analysis of Rumen, Cecum, and Rectum Microorganisms

The sample pretreatment and on-machine testing of rumen, cecum, and rectum contents were conducted by Shanghai Majorbio Bio-pharm Technology Co., Ltd (Shanghai, China). The aliquoted and preserved samples of these contents underwent total genomic DNA extraction using the sodium dodecyl sulfate (SDS) method. Subsequently, target regions were PCR-amplified with primers containing specific barcode labels, and the amplification products were verified through agarose gel electrophoresis. The PCR products were accurately quantified using the QuantiFluor™-ST fluorescence quantification system and proportionally mixed according to sequencing requirements. During the library construction phase, Illumina sequencing adapter sequences were introduced via a secondary PCR amplification, and the products were purified and recovered through gel purification. The Illumina sequencing platform employs bridge PCR amplification to generate DNA clusters, followed by sequencing via synthesis using reversible terminator chemistry, with fluorescently labeled dNTPs being used for base identification. Bioinformatic analysis is completed through OTU clustering and taxonomic annotation, including the calculation of diversity indices and the analysis of the community structure. Bioinformatic analysis was conducted using the cloud platform of Majorbio Bio-pharm Technology Co., Ltd. All data analyses were performed on the biological cloud platform (https://cloud.majorbio.com). Alpha diversity was obtained by using the mothur website (http://www.mothur.org/wiki/Calculators, accessed on 20 March 2025.) and calculating the Chao1 index, Shannon index, etc. PCoA (principal coordinate analysis) based on the Bray-Curtis distance algorithm was used to test the similarity of the microbial community structure among samples, and it was combined with a PERMANOVA non-parametric test to analyze whether the differences in the microbial community structure among sample groups were significant. A one-way analysis of variance was used to determine the bacterial groups with significant differences in horizontal abundance among different groups.

### 2.4. Metabonomic Analysis of Rumen Contents

The pre-processing of rumen content samples, on-machine detection, mass spectrometry detection, and raw data processing were completed by Wuhan Maiwei Metabolic Biotechnology Co., Ltd. (Wuhan, China). The processed data underwent pattern recognition and Pareto scaling pre-processing using SIMCA-P14.1 software. Multivariate statistical analysis was conducted on the processed data, including principal component analysis (PCA) and orthogonal partial least squares discriminant analysis (OPLS-DA). An expression model was constructed, and permutation testing was performed on the data within the model, while the variable importance in projection (VIP) was calculated based on the OPLS-DA model. Subsequently, univariate statistical analysis was conducted on the data, including fold change (FC) and *t*-test analyses. Using the criteria of FC ≥ 2 or ≤ 0.5, VIP > 1, and *p* < 0.05, differential metabolites were screened. The significant differential metabolites identified were subjected to cluster analysis and metabolic pathway analysis.

### 2.5. Data Statistics and Analysis

The experimental data were statistically analyzed using Excel 2019, and variance analysis was conducted using SPSS Statistics 22.0 software. A *p*-value ≤ 0.05 was considered significant, while *p* > 0.05 was regarded as not significant. The rate of false positives was controlled using the FDR method.

## 3. Results

### 3.1. Analysis of Microbial Differences in Rumen, Cecum, and Rectum of Tan Sheep with High and Low FCRs

#### 3.1.1. Analysis of Microbial Differences in the Rumen of Tan Sheep with High and Low FCRs

**Figure 1:**
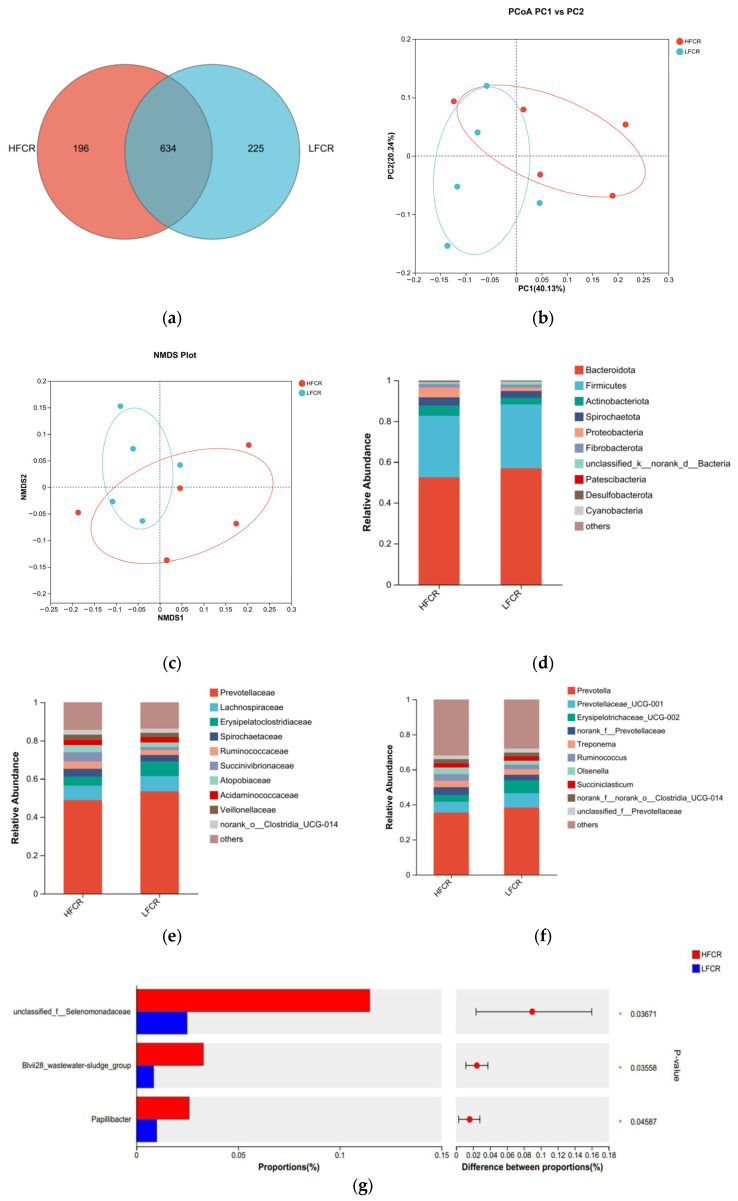
(**a**) OTU Venn analysis; (**b**,**c**) PCoA and NMDS plots; (**d**–**f**) relative abundance at phylum, family, and genus levels; (**g**) plot of differential genera among microbes.

Paired-end sequencing was conducted on the microbial community DNA fragments extracted from the rumen fluid of high- and low-FCR Tan sheep. Following screening and filtering, 1,856,680 raw reads were generated from the rumen fluid, with an average of 64,023 raw reads per sample. An analysis of the rumen microbiota in the high-FCR (HFCR) and low-FCR (LFCR) groups of Tan sheep revealed 196 unique differential operational taxonomic units (OTUs) in the HFCR group and 225 unique differential OTUs in the LFCR group, in addition to 643 OTUs shared by both groups (Figure 1a). Alpha diversity index analysis indicated no significant differences in the total number of classifications, diversity, evenness, the number of OTUs, or the total number of species among the rumen microbiota groups (*p* > 0.05) (Table 2). Principal coordinate analysis (PCoA) and non-metric multidimensional scaling (NMDS) demonstrated some separation between the two groups, although not significant, thus indicating minor differences in the composition of the rumen microbial structure between the HFCR and LFCR groups of Tan sheep (Figure 1b,c). A further comparison of the classification of rumen microbes in the HFCR and LFCR groups revealed similarities in their microbial structures. Investigations at the phylum level identified seven dominant phyla present in the rumen of Tan sheep from both the HFCR and LFCR groups. Among these, Bacteroidetes exhibited the highest relative abundance, exceeding 50%, ranking first, while Firmicutes ranked second with a relative abundance greater than 30% (Figure 1d). Analysis at the family level indicated that the relative abundance of Prevotellaceae in the rumen of Tan sheep in both the HFCR and LFCR groups surpassed 40%, making it the most abundant family (Figure 1e). At the genus level, the relative abundance of Prevotella in the rumen of Tan sheep from both the HFCR and LFCR groups exceeded 30%, thus representing the highest proportion (Figure 1f). An analysis of the top 15 most abundant microorganisms in the rumen fluid of Tan sheep from the HFCR and LFCR groups identified three genera that were significantly different: unclassified_f__Selenomonadaceae, Blvii28_wastewater-sludge_group, and Papillibacter (*p* < 0.05) (Figure 1g).

#### 3.1.2. Analysis of Microbial Differences in the Cecum of Tan Sheep with High and Low FCRs

**Figure 2:**
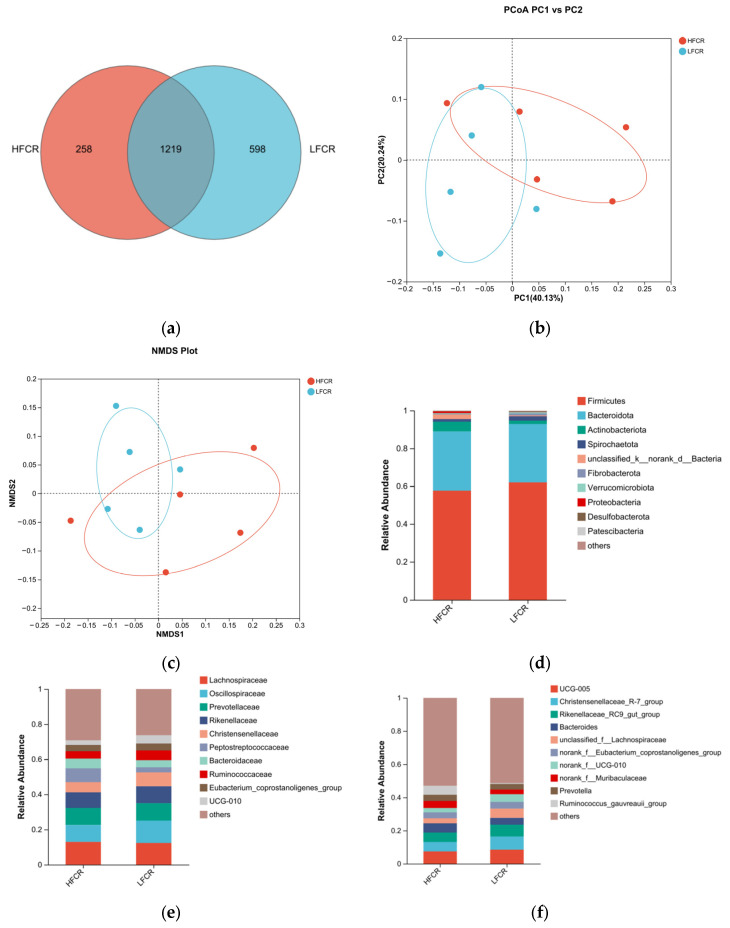

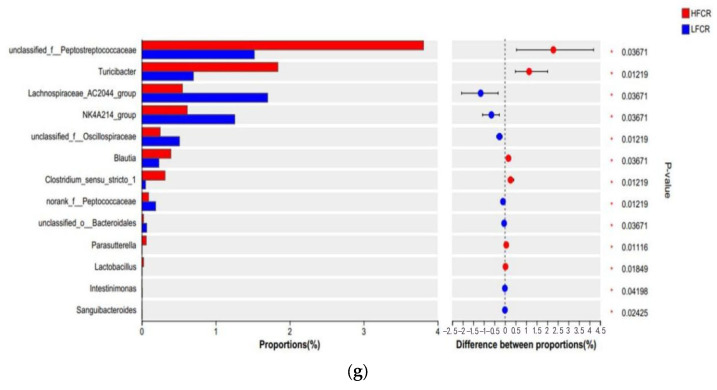
(**a**) OTU Venn analysis; (**b**,**c**) PCoA and NMDS plots; (**d**–**f**) relative abundance at phylum, family, and genus levels; (**g**) plot of differential genera among microbes.

Paired-end sequencing was conducted on the DNA fragments of the microbial community in the cecum of Tan sheep. After screening and filtering, 1,885,780 raw reads were generated from the cecum, with an average of 65,025 raw reads per sample. An analysis of the cecal microbiota in the HFCR and LFCR groups of Tan sheep revealed that, in addition to the 1219 operational taxonomic units (OTUs) shared by both groups, there were 258 unique differential OTUs in the HFCR group and 598 unique differential OTUs in the LFCR group (Figure 2a). Alpha diversity index analysis indicated that the Ace and Chao index values of the cecal microbiota in the HFCR group were significantly lower than those in the LFCR group (*p* < 0.05) (Table 3). Principal coordinate analysis (PCoA) and non-metric multidimensional scaling (NMDS) showed minor differences in the structural composition of cecal microbiota samples between the HFCR and LFCR groups of Tan sheep (Figure 2b,c). At the phylum level, both the HFCR and LFCR groups exhibited five dominant phyla in the cecum, primarily composed of Firmicutes and Bacteroidetes, with Firmicutes being the most abundant, accounting for over 50% of the relative abundance, while Bacteroidetes ranked second, with a relative abundance of approximately 30% (Figure 2d). At the family level, the HFCR and LFCR groups demonstrated higher relative abundances of Lachnospiraceae, Oscillospiraceae, and Prevotellaceae in the cecal microbiota, with each being around 10% (Figure 2e). At the genus level, the analysis revealed that both the HFCR and LFCR groups exhibited the highest relative abundance of UCG-005 in the cecal microbiota (Figure 2f). The analysis of differential bacterial genera was conducted by selecting the top 15 most abundant microorganisms in the cecum of Tan sheep from the HFCR and LFCR groups. The results revealed 13 genera that were significantly different (*p* < 0.05), including unclassified_f__Peptostreptococcaceae, Turicibacter, Lachnospiraceae_AC2044_group, NK4A214 group, unclassified_f__Oscillospiraceae, Blautia, Clostridium sensu stricto 1, norank_f__Peptococcaceae, unclassified o Bacteroidales, Parasutterella, Lactobacillus, Intestinimonas, and Sanguibacteroides (Figure 2g).

#### 3.1.3. Analysis of Microbial Differences in the Rectum of Tan Sheep with High and Low FCRs

**Figure 3:**
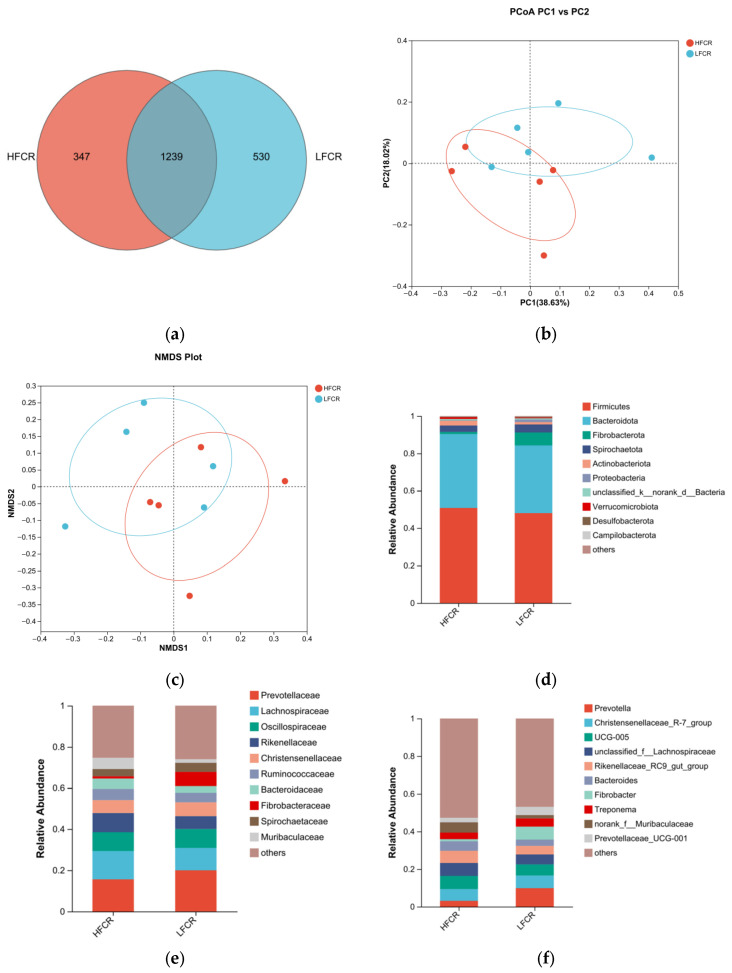

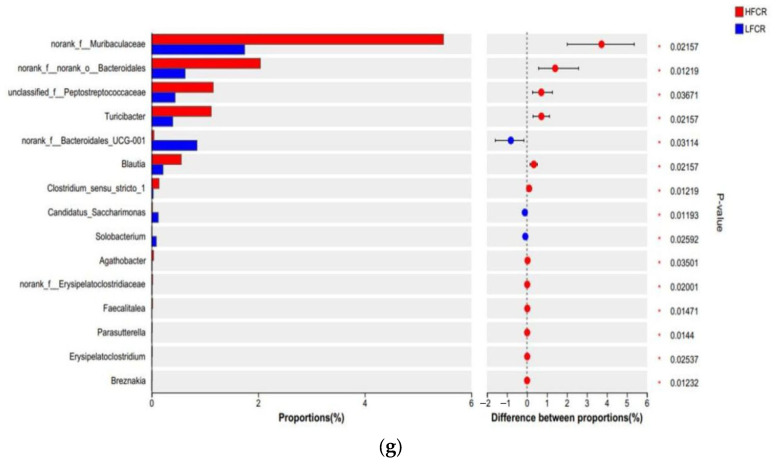
(**a**) OTU Venn analysis; (**b**,**c**) PCoA and NMDS plots; (**d**–**f**) relative abundance at phylum, family, and genus levels; (**g**) plot of differential genera among microbes.

Paired-end sequencing was performed on the DNA fragments of the microbial community in the rectum of Tan sheep. After screening and filtering, 2,196,044 raw reads were generated from the rectum, with an average of 75,725 raw reads per sample. The analysis of the rectal microbiota of Tan sheep in the HFCR and LFCR groups revealed that, in addition to the 1239 operational taxonomic units (OTUs) shared by both groups, there were 347 unique differential OTUs in the HFCR group and 530 unique differential OTUs in the LFCR group (Figure 3a). Information on species richness and diversity within the community was obtained through alpha diversity index analysis. In this study, no significant differences were observed in the total number of classifications, diversity and evenness, the number of OTUs, or the total number of species among the rectal microbial groups (*p* > 0.05) (Table 4). Principal coordinate analysis (PCoA) and non-metric multidimensional scaling (NMDS) revealed that the rectal microbial communities of Tan sheep in the HFCR and LFCR groups exhibited some differences, although these were not statistically significant (Figure 3b,c). At the phylum level, there were five dominant phyla in the rectum of Tan sheep in both the HFCR and LFCR groups, with Firmicutes having the highest relative abundance, exceeding 50%, followed by Bacteroidetes with a relative abundance of approximately 30% (Figure 3d). At the family level, the relative abundances of Oscillospiraceae, Lachnospiraceae, and Prevotellaceae in the rectal microbiota of both the HFCR and LFCR groups were relatively high at around 10% (Figure 3e). At the genus level, the relative abundance of Prevotella in the rectal microbiota of both the HFCR and LFCR groups was the highest (Figure 3f). The analysis of differential microbial genera was conducted by selecting the top 15 microorganisms in terms of rectal abundance from the HFCR and LFCR groups of Tan sheep. The results revealed significant differences (*p* < 0.05) in 15 microorganisms: norank_f__Muribaculaceae, norank_f__norank_o__Bacteroidales, unclassified_f__Peptostreptococcaceae, Turicibacter, norank_f__Bacteroidales_UCG-001, Blautia, Clostridium_sensu_stricto_1, Candidatus_Saccharimonas, Solobacterium, Agathobacter, norank_f__Erysipelatoclostridiaceae, Faecalitalea, Parasutterella, Erysipelatoclostridium, and Breznakia (Figure 3g).

### 3.2. Analysis of Correlation Between Differential Microbes in Rumen, Cecum, and Rectum of Tan Sheep with High and Low FCRs

**Figure 4:**
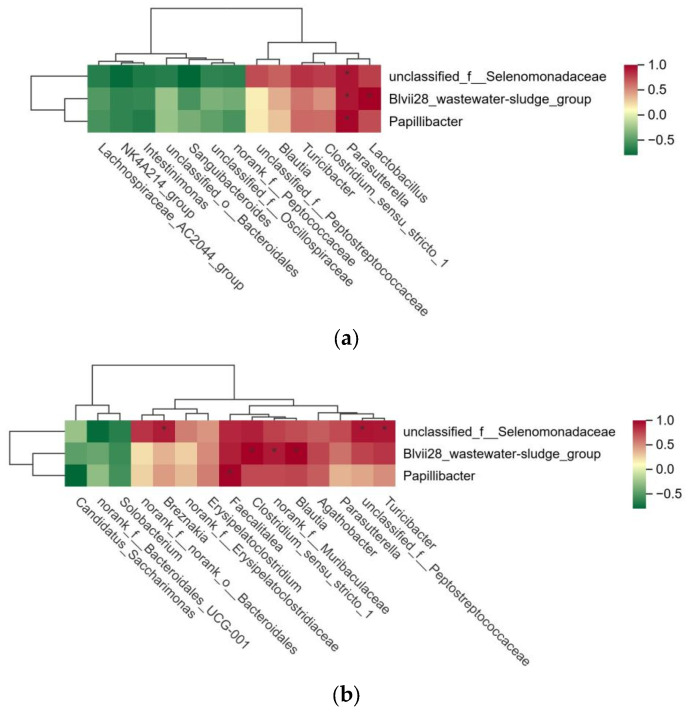
(**a**) Analysis of microbial correlation between rumen and cecum; (**b**) analysis of microbial correlation between rumen and rectum.

Based on the successful identification of significantly different microbial communities in the rumen, cecum, and rectum of high- and low-FCR Tan sheep, a correlation analysis of these microbial communities was further conducted. The results indicated that unclassified_f__Selenomonadaceae, Blvii28_wastewater-sludge_group, and Papillibacter in the rumen were significantly positively correlated with Parasutterella in the cecum (*p* < 0.01). Additionally, Blvii28_wastewater-sludge_group in the rumen showed a significant positive correlation with Lactobacillus in the cecum (*p* < 0.01) (Figure 4a). Furthermore, unclassified_f__Selenomonadaceae in the rumen exhibited a highly significant positive correlation (*p* < 0.01) with Turicibacter, unclassified_f__Peptostreptococcaceae, and Breznakia in the rectum. Blvii28_wastewater-sludge_group in the rumen also displayed a highly significant positive correlation (*p* < 0.01) with Blautia, norank_f__Muribaculaceae, and Clostridium_sensu_stricto_1 in the rectum. Lastly, Papillibacter in the rumen demonstrated a highly significant positive correlation (*p* < 0.01) with Faecalitalea in the rectum (Figure 4b).

### 3.3. Analysis of Metabolite Differences in the Rumen of Tan Sheep with High and Low FCRs

**Figure 5:**
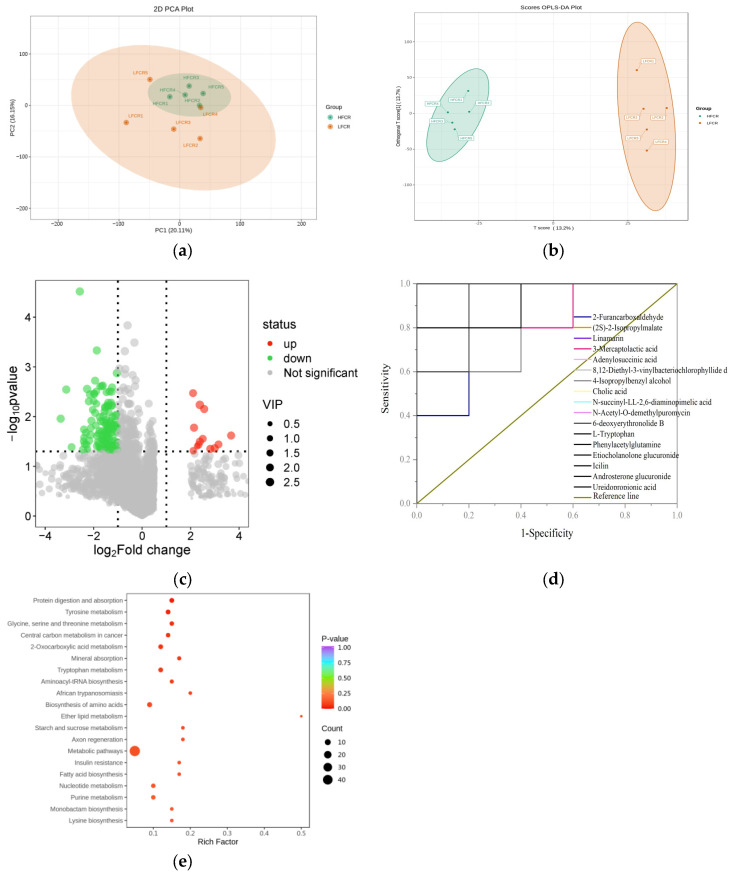
(**a**,**b**) PCA and OPLS-DA plots; (**c**) volcano plot of differential metabolites; (**d**) ROC curve of differential metabolites; (**e**) metabolic pathways of differential metabolites.

The PCA results indicated an intersection between the HFCR and LFCR groups, which was accompanied by a discernible separation trend, thus suggesting the presence of differential metabolites. Furthermore, the samples within the HFCR group exhibited strong clustering, reflecting high similarity in metabolite composition (Figure 5a). The OPLS-DA analysis demonstrated a clear distinction between the HFCR and LFCR groups, which were in separate regions. Each group’s samples were tightly clustered, with a significant distance between the groups, thus indicating robust stability and predictive capability, effectively distinguishing the rumen metabolite composition between the HFCR and LFCR groups of Tan sheep (Figure 5b). Utilizing the criteria of FC ≥ 2 or ≤ 0.5, VIP > 1, and *p* < 0.05, 297 differential metabolites were identified between the HFCR and LFCR groups in the rumen of Tan sheep. This included 46 amino acids and their metabolites; 46 benzene derivatives; 39 organic acids and their derivatives; 34 heterocyclic compounds; 33 aldehydes, ketones, and esters; and 99 other types of metabolites. In comparison to the HFCR group, the LFCR group exhibited 261 downregulated and 36 upregulated differential metabolites in the rumen (Figure 5c). The ROC curve analysis identified 17 significantly different metabolites, including 2-Furancarboxaldehyde, (2S)-2-isopropylmalate, Linamarin, and 3-Mercaptolactic acid, in the rumen of Tan sheep across the LFCR and HFCR groups (Figure 5d). An Area Under the Curve (AUC) greater than 0.8 indicates that the differential metabolite possesses excellent specificity. When arranged by ascending *p*-value, three significantly different metabolites (*p* < 0.01) were identified between the HFCR and LFCR groups of Tan sheep: 8,12-Diethyl-3-vinylbacteriochlorophyllide d, Phenylacetylglutamine, and Androsterone glucuronide (Table 5). The KEGG results indicated that the differentially expressed metabolites in the rumen of Tan sheep, when comparing the HFCR and LFCR groups, were primarily enriched in metabolic pathways, including those for protein digestion and absorption, tyrosine metabolism, and glycine, serine, and threonine metabolism (Figure 5e).

### 3.4. Analysis of Correlation Between Rumen Microbes and Metabolites in Tan Sheep with High and Low FCRs

**Figure 6:**
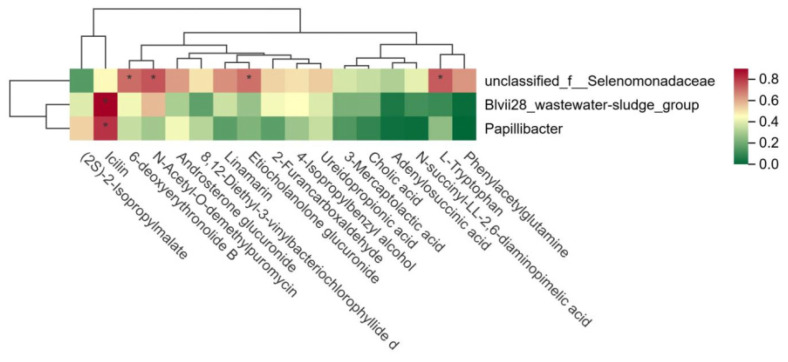
Correlation between rumen microorganisms and rumen metabolites. * indicates a significant correlation between the two microorganisms (*p* < 0.01).

A further analysis of the relationship between rumen microbiota and metabolites in the HFCR and LFCR groups of Tan sheep revealed that unclassified_f__Selenomonadaceae exhibited significant positive correlations with L-tryptophan, Etiocholanolone glucuronide, N-acetyl-O-demethylpuromycin, and 6-deoxyerythronolide B (*p* < 0.05). Additionally, Blvii28_wastewater-sludge_group and Papillibacter showed significant positive correlations with Icilin (*p* < 0.05) (Figure 6).

## 4. Discussion

Changes in the gastrointestinal microbial community significantly affect the feed utilization efficiency of ruminants [17,18,19,20]. This study found that the dominant microbial communities in the rumen of Tan sheep with high, medium, and low FCRs are generally similar, with Bacteroidetes and Firmicutes ranking as the top two in terms of relative abundance. Tom et al. discovered that the rumen microbial communities of Holstein cows and Jersey cows are primarily composed of phyla such as Bacteroidetes [21], which largely aligns with our findings in the rumen of Tan sheep, thus indicating that Bacteroidetes and Firmicutes are the dominant microbial communities in the rumen of ruminants. This study further observed that the relative abundances of Prevotellaceae and Prevotella in the rumen of low-FCR Tan sheep were 4.64% and 2.87% higher, respectively, than those in high-FCR Tan sheep. Moreover, the experimental results of Bo Trabi et al.’s study showed that the FCR of Hu sheep in the experimental group with higher Prevotella abundance in the rumen was greater than that of the control group with lower Prevotella abundance [22], which is largely consistent with our findings. Previous studies have shown that Prevotella are rich in glycoside hydrolase and peptidase activities and can actively participate in the fermentation and degradation of carbohydrates and the hydrolysis of proteins in the rumen. Moreover, the abundance of Prevotella is positively correlated with the expression of genes involved in fatty acid metabolism and other aspects in rumen epithelial tissue. These mechanisms work together to help improve the utilization efficiency of the energy of the host [23,24,25]. Furthermore, this study observed that the three significantly different genera present in the rumen of high- and low-FCR Tan sheep—unclassified_f__Selenomonadaceae, Blvii28_wastewater-sludge_group, and Papillibacter—were all downregulated in the rumen of low-FCR Tan sheep. Gao et al. suggested that the reduction in the abundance of unclassified_f__Selenomonadaceae in the rumen may enhance fiber degradation capacity by decreasing substrate competition with Prevotella, while the accumulation of its metabolic product, propionate, may inhibit the proliferation of inefficient bacterial genera such as Blvii28_wastewater-sludge_group [26]. The proliferation of inefficient genera, such as Blvii28_wastewater-sludge_group, may competitively inhibit the activity of cellulolytic enzymes [27,28]. In this study, we observed that the abundance of Papillibacter rumen microorganisms significantly decreased when the feed utilization efficiency of Tan sheep increased. This finding is consistent with the results obtained from research on other ruminants. Mao et al. found that the changes in bacterial genera such as Papillibacter in the rumen of dairy cows are closely related to the rumen acidification environment. Under high-energy diet conditions, the rumen pH of dairy cows will decrease, which then causes a significant reduction in rumen Papillibacter abundance [29]. In a study on calves, Xie et al. observed that antibiotic treatment significantly reduced the abundance of Papillibacter in the feces of both diarrheal and non-diarrheal calves [30]. Notably, although the physiological stages and intervention measures of different ruminants vary, the abundance of Papillibacter is associated with the trend in feed utilization efficiency. In the future, the specific function of this bacterium in carbohydrate metabolism can be further analyzed. Additionally, the metabolites of Papillibacter can interfere with the absorption of short-chain fatty acids by rumen epithelial cells [31]. Therefore, we speculate that the synergistic effects of these three factors may negatively impact the feed utilization efficiency of Tan sheep.

The cecal microbiota, as the primary site of hindgut fermentation in ruminants, plays a crucial role in the secondary degradation of dietary fiber and the synthesis of short-chain fatty acids [32]. In contrast, the rectum is primarily involved in water absorption and residue excretion, exhibiting relatively diminished microbial activity [33]. This study found that the cecal microbiota of high- and low-FCR Tan sheep was dominated by Firmicutes at the phylum level, which is consistent with the findings of Gong et al., as Firmicutes primarily function in degrading fibrous materials and promoting fat deposition in the host’s intestine [10]. Notably, the relative abundance of Firmicutes in low-FCR Tan sheep was 4.31% higher than that in the high-FCR group. We speculate that the reduction in the FCR in Tan sheep is also attributed to the increased abundance of Firmicutes in the cecum. As a key functional genus within this phylum, Christensenellaceae_R-7_group has been shown to enhance the activity of lignocellulose hydrolase [34]. This study found that its relative abundance in low-FCR Tan sheep was 2.14% higher than that in high-FCR Tan sheep, thus suggesting that Christensenellaceae_R-7_group plays a significant role in fiber degradation in low-FCR Tan sheep. Furthermore, the relative abundance of Prevotella in the rectum of low-FCR Tan sheep showed an increasing trend compared to that in their high-FCR counterparts. Research by Yin et al. indicated that the intestinal content of Prevotella in lambs with high feed efficiency was greater than that in those with low feed efficiency [35], which aligns with the findings of this study, thus suggesting that Prevotella is associated with an improvement in intestinal metabolic efficiency. Additionally, this study found that the relative abundance of norank_f__Bacteroidales_UCG-001 in the rectum of Tan sheep with a low FCR was significantly higher than that in Tan sheep with a high FCR. In their analysis of rumen microbiota in goats, Tian et al. observed that the abundance of norank_f__Bacteroidales_UCG-001 was greater in the rumen of goats with high feed utilization efficiency compared to those with low feed utilization efficiency [36]. We speculate that norank_f__Bacteroidales_UCG-001 may also play a role in promoting feed utilization efficiency in Tan sheep.

This study also found that unclassified_f__Selenomonadaceae, Blvii28_wastewater-sludge_group, and Papillibacter in the rumen were significantly positively correlated with Parasutterella in the cecum. Additionally, Blvii28_wastewater-sludge_group exhibited a significant positive correlation with Lactobacillus. Unclassified_f__Selenomonadaceae in the rumen was significantly positively correlated with Turicibacter, unclassified_f__Peptostreptococcaceae, and Breznakia in the rectum. Furthermore, Blvii28_wastewater-sludge_group showed a significant positive correlation with Blautia, norank_f__Muribaculaceae, and Clostridium_sensu_stricto_1, while Papillibacter was significantly positively correlated with Faecalitalea. Unclassified_f__Selenomonadaceae in the rumen appeared to be more dependent on easily fermentable carbohydrates, and the high-fiber diet of Tan sheep may inhibit their growth [37,38]. Lactobacillus and norank_f__Muribaculaceae are both probiotics, which means that they may be related to the secondary degradation of dietary fiber and the generation of short-chain fatty acids [39,40]. In this study, the relative abundance of Lactobacillus and norank_f__Muribacaceae increased, while the feed utilization efficiency decreased. We speculate that this contradictory phenomenon may be caused by competition with other gut microbiota. However, there is relatively little research on Blvii28_wastewater-sludge_group in ruminants. Considering the potential pathogenicity of Blvii28_wastewater-sludge_group, we hypothesize that it may be a potentially harmful bacterial genus, and sheep with high feed utilization efficiency may only form niche competition inhibition against this genus through the proliferation of dominant beneficial bacteria. Parasutterella, a gut symbiont closely related to host metabolism, primarily colonizes the hindgut, and variations in its abundance may be closely linked to the rumen microbiota [41]. This study found that the abundance changes in Parasutterella in the appendix were significantly positively correlated with the abundance of key bacterial genera, such as unclassified_f__Selenomonadaceae, in the rumen. This suggests that the rumen microbiota may influence fermentation products such as short-chain fatty acids and indirectly regulate the postintestinal environment and the colonization and abundance of symbiotic bacteria such as Parasutterella within it. We speculate that the decrease in the abundance of Selenomonadaceae exerts an inhibitory effect on butyrate metabolism, thus leading to a reduction in the abundance of Parasutterella in the cecum. The enrichment of Faecalitalea in the rectum indicates a lower abundance of beneficial bacteria, such as Prevotella, in the host’s gut [42], thus validating the previous hypothesis that Papillibacter interferes with the transport of short-chain fatty acids in rumen epithelial cells.

Metabolomics is a crucial approach for elucidating the nutritional metabolic mechanisms in ruminants [43]. For the first time, this study identified three key differential metabolites—Phenylacetylglutamine, 8,12-Diethyl-3-vinylbacteriochlorophyllide d, and Androsterone glucuronide—in the rumen of high- and low-FCR Tan sheep, all of which were downregulated in the rumen of low-FCR Tan sheep. Brusilow et al. found that the complete utilization of Phenylacetylglutamine is advantageous for nitrogen degradation [44]. The nitrogen degradation capacity of the rumen in Tan sheep can influence protein breakdown. Additionally, Phenylacetylglutamine is the end product of nitrogen clearance. In this study, the accumulation of Phenylacetylglutamine in the rumen of low-FCR Tan sheep reflects that the nitrogen source in the feed of low-FCR Tan sheep was not efficiently converted into microbial protein, thus resulting in nitrogen waste [45]. Furthermore, some studies have identified Phenylacetylglutamine as a disease marker [46,47], which may have adverse effects on the host, thereby indicating that high-FCR Tan sheep could be at risk for potential diseases. KEGG pathway analysis revealed that the most significant enrichment pathways were those for protein digestion and absorption, and this result was highly consistent with the findings of previous studies wherein the mechanism by which protein metabolism regulates feed efficiency was explored. Li et al. confirmed this using a high-protein diet, as protein digestion and absorption were optimized, the lamb nitrogen deposition rate increased, and a higher total protein content was obtained [48]. This is corroborated by the reduction in the rumen metabolite Phenylacetylglutamine and the reduction in nitrogen degradation in low-FCR Tan sheep in this study, thus suggesting that the activation of this pathway may enhance the host’s nitrogen retention efficiency by reducing nitrogen loss. In a study by Benhissi et al., the supply of microbial protein in the experimental group increased by 37%, and in a study by Zeng et al., the methane emissions per unit weight gain of low- and medium-RFI sheep decreased by 9%. These phenomena jointly point to the possibility that the protein digestion and absorption pathways may improve nitrogen conversion efficiency and reduce the energy consumed for host maintenance. The reduction in pyruvate metabolic pathway metabolites in low-FCR Tan sheep in this study further supports the existence of a more efficient energy allocation pattern in this group [49,50].

We also found that unclassified_f__Selenomonadaceae in the rumen exhibited a significant positive correlation with metabolites such as L-tryptophan. This positive association may reflect the role of unclassified_f__Selenomonadaceae in consuming tryptophan to produce indole derivatives, thereby reducing the host’s utilization of essential amino acids [26]. According to the metabolomic findings, L-tryptophan was primarily enriched in the amino acid metabolic pathway. Sinha et al. discovered that fiber could inhibit the balance of tryptophan metabolic flow by regulating microbial metabolism [51]. In conjunction with the previously mentioned analysis indicating a decrease in the abundance of the rumen microbe unclassified_f__Selenomonadaceae, Hu et al. discovered that L-tryptophan flow to the serotonin pathway will enhance sheep production performance, and higher serotonin can promote sheep feed efficiency [52] and significantly reduce the stress response. To reduce energy consumption, more nutrients are needed to increase the deposition rate [53]. In this study, the level of L-tryptophan in low-FCR Tan sheep was relatively low, which might be related to its more efficient flow to the serotonin pathway to optimize feed utilization. We speculate that this microbe may regulate the involvement of L-tryptophan in the amino acid metabolic pathway by inhibiting the conversion of tryptophan to indole. Amino acid metabolism not only influences the rumen function of ruminants but also supports the normal metabolism and functions of their bodies [54,55]. In this study, the low level of L-tryptophan observed in low-FCR Tan sheep may be associated with its rapid conversion into immune-modulating substances such as serotonin. Efficient tryptophan metabolism ensures adequate levels of melatonin and serotonin, which enhance the immune capacity of sheep [56]. Additionally, the reduction in metabolites such as 2-isopropylmalate in pyruvate metabolism suggests a more streamlined and efficient energy metabolism pathway [57,58,59].

## 5. Conclusions

The composition of the gastrointestinal microbial community in Tan sheep is significantly associated with feed utilization efficiency. Compared with high-FCR Tan sheep, the rumen of low-FCR Tan sheep has a significantly reduced abundance of microorganisms such as unclassified_f__Selenomonadaceae, and metabolites such as Phenylacetylglutamine are significantly reduced as well, while other metabolites such as Glycyl-L-tyrosine increased significantly. Microorganisms such as Lachnospiraceae_AC2044_group in the cecum increased significantly, and unclassified_f__Peptostreptococcaceae decreased significantly. The number of norank_f__Bacteroidales_UCG-001 in the rectum increased significantly, while the number of norank_f__Muribaculaceae and other microorganisms decreased significantly. Some microorganisms, such as unclassified_f__Selenomonadaceae in the rumen of high- and low-FCR Tan sheep, are closely related to microorganisms such as Parasutterella in the cecum and Turicibacter in the rectum, and they are also closely related to metabolites such as L-tryptophan in the rumen. This study analyzed the key role of gastrointestinal microorganisms in the digestion and absorption of feed in Tan sheep, thus providing a preliminary foundation for further research on the regulation of feed utilization in ruminants via microorganisms.

## Figures and Tables

**Table 1:** Composition and nutrient levels of basal diets.

Items	Content %
Ingredients	
Corn straw	20.00
Corn	32.00
Molasses	4.00
Soybean meal	6.00
Cotton meal	8.00
Corn bran	15.30
Corn germ meal	11.00
Limestone	1.20
NaCl	0.50
Premix ^1^	2.00
Total	100.00
Nutrient levels ^2^	
ME/(MJ/kg)	9.93
CP	13.74
EE	2.74
NDF	35.99
ADF	20.34
Ca	0.71
TP	0.31

^1^ Per kilogram of diet, the premix provides the following: 250,000 IU of vitamin A, 375 IU of vitamin E, 100,000 IU of vitamin D, 850 mg of iron, 800 mg of copper, 750 mg of zinc, 750 mg of manganese, 25 mg of selenium, 50 mg of iodine, and 10 mg of cobalt. ^2^ Metabolizable energy is calculated according to NY/T 816–2004, and the rest of the values are measured.

**Table 2:** Alpha diversity index table.

Item	HFCR	LFCR	*p*-Value
Shannon	4.03 ± 0.22	3.95 ± 0.2	0.560
Simpson	0.04 ± 0.01	0.05 ± 0.02	0.247
Ace	533.36 ± 58.21	536.75 ± 43.68	0.920
Chao	522.12 ± 58.38	542.18 ± 40.52	0.546

**Table 3:** Alpha diversity index table.

Item	HFCR	LFCR	*p*-Value
Shannon	4.71 ± 0.63	5.28 ± 0.21	0.089
Simpson	0.03 ± 0.03	0.01 ± 0.004	0.239
Ace	900.45 ± 167.45a	1155.71 ± 59.77b	0.012
Chao	889.51 ± 156.297a	1130.68 ± 55.17b	0.012

Different letters (a,b) in the same row indicate significant differences (*p* < 0.05), while no letters indicate no significant differences (*p* > 0.05).

**Table 4:** Alpha diversity index table.

Item	HFCR	LFCR	*p*-Value
Shannon	4.95 ± 0.36	4.93 ± 0.54	0.925
Simpson	0.03 ± 0.03	0.02 ± 0.02	0.869
Ace	979.82 ± 133.66	1070.51 ± 164.26	0.366
Chao	959.69 ± 130.88	1062.47 ± 146.64	0.276

**Table 5:** Differential metabolites in the rumen of the high- and low-FCR groups.

Metabolites	VIP	*p*-Value	FC
Phenylacetylglutamine	2.06	0.002	0.30
8,12-Diethyl-3-vinylbacteriochlorophyllide d	2.32	0.003	0.42
Androsterone glucuronide	2.44	0.004	0.23

## Data Availability

Data are contained within the article.

## Abbreviations

The following abbreviation is used in this manuscript:

FCR	Feed Conversion Ratio
